# Intermediate Levels of Hippocampal Activity Appear Optimal for Associative Memory Formation

**DOI:** 10.1371/journal.pone.0013147

**Published:** 2010-10-01

**Authors:** Xiao Liu, Shaozheng Qin, Mark Rijpkema, Jing Luo, Guillén Fernández

**Affiliations:** 1 Centre for Cognitive Neuroimaging, Donders Institute for Brain, Cognition and Behaviour, Radboud University Nijmegen, Nijmegen, The Netherlands; 2 Institute of Psychology, Chinese Academy of Sciences (CAS), Beijing, China; 3 Graduate School, Chinese Academy of Sciences (CAS), Beijing, China; 4 Department of Neurology, Radboud University Nijmegen Medical Centre, Nijmegen, The Netherlands; 5 Department for Cognitive Neuroscience, Donders Institute for Brain, Cognition and Behaviour, Radboud University Nijmegen Medical Centre, Nijmegen, The Netherlands; University of Granada, Spain

## Abstract

**Background:**

It is well established that hippocampal activity is positively related to effective associative memory formation. However, in biological systems often optimal levels of activity are contrasted by both sub- and supra-optimal levels. Sub-optimal levels of hippocampal activity are commonly attributed to unsuccessful memory formation, whereas the supra-optimal levels of hippocampal activity related to unsuccessful memory formation have been rarely studied. It is still unclear under what circumstances such supra-optimal levels of hippocampal activity occur. To clarify this issue, we aimed at creating a condition, in which supra-optimal hippocampal activity is associated with encoding failure. We assumed that such supra-optimal activity occurs when task-relevant information is embedded in task-irrelevant, distracting information, which can be considered as noise.

**Methodology/Principal Findings:**

In the present fMRI study, we probed neural correlates of associative memory formation in a full-factorial design with associative memory (subsequently remembered versus forgotten) and noise (induced by high versus low distraction) as factors. Results showed that encoding failure was associated with supra-optimal activity in the high-distraction condition and with sub-optimal activity in the low distraction condition. Thus, we revealed evidence for a bell-shape function relating hippocampal activity with associative encoding success.

**Conclusions/Significance:**

Our findings indicate that intermediate levels of hippocampal activity are optimal while both too low and too high levels appear detrimental for associative memory formation. Supra-optimal levels of hippocampal activity seem to occur when task-irrelevant information is added to task-relevant signal. If such task-irrelevant noise is reduced adequately, hippocampal activity is lower and thus optimal for associative memory formation.

## Introduction

The integrity of the medial temporal lobe, with the hippocampus at its core, is essential for declarative memory [1]. There is growing evidence that the hippocampus plays a critical role when disparate information has to be bound together forming new memories of associations that can be used flexibly [2]–[4]. Functional imaging studies [5]–[7] indicated as well that hippocampal activity at study is predictive for subsequent associative retrieval. These and many other studies showed consistently that more hippocampal activity at study is related to better associative memory. However, biological systems behave often non-linearly exhibiting typically a bell-shape dose- or activity-effect function [8], [9]. In other words, one can assume that there are intermediate levels of hippocampal activity optimal for associative memory formation, whereas sub- and supra-optimal levels are related to less efficient memory formation. Functional imaging studies published so far appear to have tapped predominantly into sub-optimal and optimal levels of hippocampal activity related to subsequent misses and hits, respectively. However, several studies have reported a relationship between increased medial temporal lobe/hippocampal activity and memory failure [6], [10]–[12]. These negative subsequent memory effects in the medial temporal lobe have received little attention, and under what circumstances such supra-optimal levels of hippocampal activity occur is still poorly understood.

In real life, relevant information encoded into memory has to be extracted usually from irrelevant background information, which can be regarded as noise. Thus, formation of cleanly defined and discrete memory traces against a background of irrelevant information requires ambient noise reduction. When task-relevant associative information is submerged in irrelevant information, a supra-optimal level of hippocampal activity might be caused by the combination of neural correlates of task-relevant and task-irrelevant information. However, such an excess would not enable effective memory formation, at least not for discrete task-relevant associations. In contrast, if ambient noise is reduced effectively in such a noisy state, successful associative memory formation would go along with a lower, intermediate level of hippocampal activity relative to unsuccessful associative memory formation (i.e., negative subsequent memory effect), because activity related to task-irrelevant information would be reduced. Also, in contrast to the optimal level, associative memory formation in a conventional, low-noise condition fails if hippocampal activity is not high enough (sub-optimal level of hippocampal activity) and thus, a positive subsequent memory effect occurs. There appears to be initial empirical support for the notion that task-irrelevant noise leads to supra-optimal levels of hippocampal activity at encoding. Henckens and colleagues [12] found a negative subsequent memory effect in the hippocampus when subjects memorized complex pictures while being in an experimentally induced state of psychological stress. In such a state, hypervigilance might lead to task-irrelevant noise affecting hippocampal processing. However, neuromodulatores released during stress might have also other effects on hippocampal activity and thus, that study is in this regard not conclusive yet.

Therefore, in the present study we tested the hypothesis that intermediate levels of hippocampal activity are optimal for associative memory formation, while sub- and supra-optimal levels are associated with failure to form new associative memories. More specifically, we expect a negative subsequent memory effect in a high noise condition (i.e., subsequent remembered<subsequent forgotten) and a positive subsequent memory effect in a conventional low-noise condition (i.e., subsequent remembered>subsequent forgotten). To this end, we probed neural activity related to associative memory formation in a full-factorial design with the factor associative memory (association subsequently remembered versus forgotten) and the factor noise (high versus low distraction). While scanned, subjects memorized sequentially presented object-face pairs and the within-pair delay period was filled with either a simple visuo-motor control task (low distraction condition) or a working memory task (high distraction condition).

## Materials and Methods

### Ethics statement

All participants provided written informed consent in accordance with the declaration of Helsinki. This study was approved by the local ethics committee (Commissie Mensgebonden Onderzoek region Arnhem-Nijmegen, The Netherlands).

### Participants

Twenty-four healthy, right handed subjects with normal or corrected to normal vision (13 female; age 22.4±2.9 yrs) were recruited from the Radboud University Research Participation System. They reported no neurological or psychiatric history. The data of seventeen subjects were used for further analysis. Data from seven subjects were excluded: one due to failure in data acquisition; one due to failure in comprehending task instructions, and five subjects showed poor memory performance at or close to chance level (25%) in the associative memory test.

### Stimulus

Stimulus material consisted of 240 color photographs of objects and 360 color portraits (half males). The photographs of common, every day objects were selected from the Hemera Photo-Objects database (http://www.hemera.com). The portraits were color photographs of individuals from different European regions. These faces were photographed in a standardized fashion with mildly happy emotional expression, without headgear or glasses. One hundred-twenty out of 360 portraits were used as foils in the face recognition memory test, counterbalanced over subjects. All visual stimuli were presented in the center of the screen on a black background by Presentation software (www.neurobs.com).

### Task procedure

Initially, each subject went through a pre-experimental training session with two object-face pairs per condition not used in the actual experiment. The actual experiment consists of three phases:

#### Pre-scan familiarization

Since we were not interested in single-item memory for the objects (we have a single item memory measure for faces), and since task difficulty was quite high, subjects were familiarized with all objects before they went into the scanner for associative encoding. Subjects were asked to name and memorize all objects half an hour prior to MRI scanning. Objects were presented twice, random sequentially, in random order at the center of the screen, each for 2s with a 1s inter-stimulus interval.

#### Scanning phase (encoding task)

**Figure 1:**
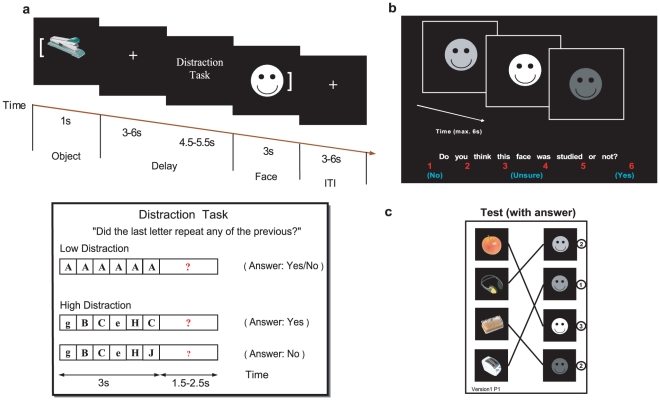
Experimental paradigm. Encoding task inside the scanner (***a***) and the post-scan memory tests (***b***, ***c***). ***a***, ***top***, In each trial, the image of an object was presented first, followed by a fixation cross, the distraction task, and the face. ***a***, ***bottom***, The distraction task was either a simple visuo-motor control task (low distraction condition) or a working memory task (high distraction condition). In both tasks, six letters were sequentially presented and subjects had to indicate whether the final letter of each sequence was identical to one of the previous five letters. ***b***, Face recognition memory test. Subjects had to make an old-new judgment on each sequentially presented face by a confidence rating on a six-point scale. ***c***, The associative memory test. Subjects had to connect the studied object-face pairs by lines and add a confidence rating. Note: in this figure, the actual face stimuli are replaced by smiley, because of unclear copyright status.

Figure 1 (a, top) shows the structure of the encoding task executed inside the scanner. Subjects were instructed to memorize 240 sequentially presented object-face pairs in which the object was always presented first for 1s. Subsequently a variable within-pair delay of 7.5 to 11.5 s followed and finally the face was presented for 3 s. The distraction task was presented at the end of the within-pair delay and directly followed by the face presentation, in order to induce clear interruption right before associations were formed. Each pair was separated by a jittered inter-pair delay of 3 to 6 s. To ensure the subjects perceived the object-face pairs as one pair, the object was presented with an open square bracket on the left to indicate opening and the face was present with a close square bracket on the right to indicate closure of the pair. The entire experiment consisted of four runs, each containing 60 pairs and lasting about 19 minutes.

Half of the within-pair delay periods were filled with a simple visuo-motor control task (low distraction condition) and the other half of the within-pair delay periods were filled with a working memory task (high distraction condition, Fig. 1a, bottom). Both distraction tasks had a match-to-sample structure, in which six letters were sequentially presented in the center of the screen for 500 ms, each with 500 ms intervals. In the simple visuo-motor control task, the capital letter “A” was presented six times. In the working memory task, six different letters (half upper case) or five different plus one repeated, final letter. Subjects were instructed to press corresponding buttons with their right index or middle finger at the end of the working memory task to show whether they detected a repeated letter, regardless of case. In the simple visuo-motor control task, they were instructed to give one random button press with their right index or middle finger at the end of each sequence.

#### Post-scan memory tests

Two memory tests were applied immediately after scan. First, a face recognition memory test in which 240 old, previously studied faces were sequentially shown on a computer screen randomly intermixed with 120 new, yet unstudied faces. Subjects were instructed to indicate whether they had seen the face before in the scanner by a confidence rating on a six-point scale (Fig. 1b). On this scale, a ‘6’ response was associated with the highest confidence for prior occurrence and a ‘1’ response with the highest confidence for a new stimulus. After completing the face recognition memory test, subjects performed an associative memory test in a paper-and-pencil manner: 240 object-face pairs were randomly assigned into 60 clusters with four pairs printed on each page. The locations of objects and faces were randomized (Fig. 1c). Subjects were instructed to connect the studied pairs by lines and to add a confidence rating indicating whether they were absolutely sure that it is the right link (1), somewhat sure (2), or just guessing/excluding (3).

### fMRI data acquisition

Whole-brain T2*-weighted images were acquired on a 1.5 T Siemens Avanto MR-scanner. Functional images were recorded using an ascending slice acquisition EPI sequence (33 axial slices, matrix 64×64, slice thickness 3.4 mm, slice gap 0.34 mm, flip-angle 90°, TR 2190 ms, TE 35 ms, voxel size 3.3×3.3×3.4 mm^3^). T1-weighted anatomical images were acquired using an MPRAGE sequence (176 sagittal slices, matrix 256×256, slice thickness 1mm, flip-angle 15°, TR 2250 ms, TE 2.95 ms, T1 = 850 ms, voxel size 1×1×1 mm^3^).

### Behavioral data analysis

All statistical tests of behavioral data were performed using SPSS (15.0, SPSS Inc, Chicago, USA). To test whether subjects discriminated successfully between old (previously studied) and new (previously unstudied) faces, recognition memory data was analyzed using a 2×6 ANOVA with the study status of the items as one factor (old versus new) and the confidence rating as the other factor (six levels). Post-hoc paired-sample t-tests were applied to test for old-new discriminability at each level of confidence. Additionally, we tested whether recognition confidence for correctly identified old faces differed between the two distraction conditions (low versus high distraction) using a 2×6 ANOVA with the factor distraction and the level of confidence.

Based on the results in the item and the associative memory tests (see Behavioral results), we further analyzed our data in a repeated measures 3×2 ANOVA. The first factor, memory status, contained three levels: Item forgotten (face and association forgotten), Association forgotten (face remembered but association forgotten), and Association remembered (both face and association remembered). The second factor was the distraction condition (low versus high distraction). In this way we investigated differences between memory status, distraction conditions, and their interactions.

### fMRI data analysis

Image preprocessing and statistical analysis were performed using Statistical Parametric Mapping (SPM5, www.fil.ion.ucl.ac.uk) which ran under MATLAB 7.2 (Math Works, Inc). The first five EPI volumes of each subject were discarded to allow for T1 equilibration. The images were preprocessed using the following processing steps: realignment to correct for head motion, coregistration of the mean of the functional images to each subject's high resolution T1-weighted image, slice time correction, spatial normalized to a common stereotactic space defined by SPM MNI152 (Montreal Neurological Institute) T1 template, resampling into 3×3×3 mm voxels, and finally smoothing with an isotropic 3D Gaussian kernel with 8 mm FWHM. The data were statistically analyzed in the framework of the General Linear Model and Statistical Parametric Mapping [13].

For the first level analysis we specified a general linear model in which events were sorted into several regressors as a function of the trial component, the distraction condition, and subsequent memory. Firstly, the presentation of each object was included as an event of 1 s. The distraction period was modeled into two regressors (low and high distraction) for the period of the letter sequence (3 s). Since our main interest is the neural activity related to memory formation in the different conditions, the face presentations were included in the model as 3 s events and sorted into six different regressors according to the same six bins that we defined in the 3×2 design in the behavioral data analysis. All remaining trials were included into an extra condition of no interest. Fixation periods were not modeled and used as a low level baseline. All regressors were convolved with the canonical Hemodynamic Response Function (HRF) in SPM5. In addition, the realignment parameters were separately modeled to account for movement-related variability.

Contrast images generated in the first level analysis were submitted to a group level full factorial 3×2 ANOVA with a factor memory status (Item forgotten, Association forgotten, and Association remembered) and a factor distraction (low and high). To assess the brain activity during the distraction period, we contrasted the high and low distraction conditions at the onset of letter sequence. All other statistical tests were onset to face presentation. The non-associative memory effect was tested by contrasting Association forgotten trials with Item forgotten trials. To explore the subsequent associative memory effect and its interaction with the distraction condition, we contrasted Association remembered trials with Association forgotten ones, and additionally explored the interaction between memory status (Association remembered and Association forgotten) and distraction condition (low and high). Based on our hypothesis for the hippocampus, we applied a small volume correction (SVC) to the activated brain regions found in the associative memory contrast and the interaction using the anatomical automatic labeling template of the bilateral hippocampus (WFU PickAtlas toolbox in SPM). Significant interactions were further explored using post-hoc t-tests to reveal the differences between the separate conditions. In these t-tests the peak voxel of the interaction was assessed at the same statistical threshold as the interaction.

Beta values from significantly activated regions were extracted using MarsBaR (marsbar.sourceforge.net) for visualization purposes. All fMRI analyses in this study were thresholded at *p* (uncorrected)<0.001, unless otherwise specified.

## Results

### Behavioral results

Subjects gave in 89.00±5.3% (Mean ± SD) of trials correct responses to the working memory task (high distraction condition). Reaction times in the simple visuo-motor control task (low distraction condition) were shorter than in the working memory task (paired-samples t test, 0.57±0.13 relative to 0.78±0.16, *t_(17)_* = −8.29, *p*<0.001). As intended, subjects were well able to do the two distraction tasks, but the high distraction task was substantially more difficult than the simple visuo-motor control task.

**Figure 2:**
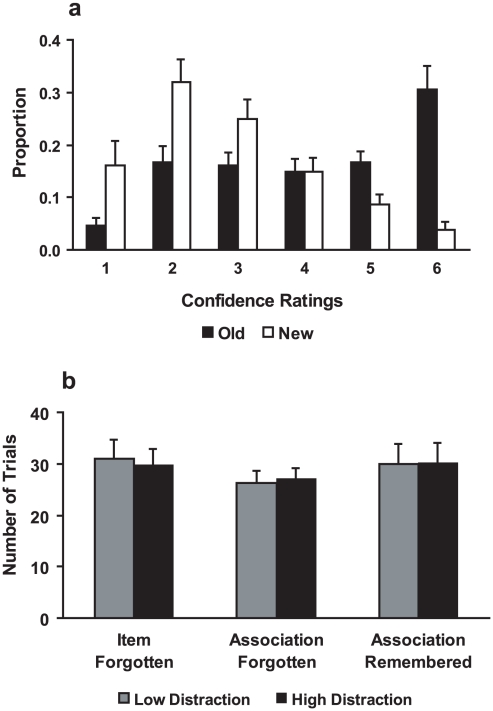
Behavioral performance. ***a***, Behavioral results of the face recognition memory test. Confidence ratings range from ‘1’ (absolutely sure that the face is new) to ‘6’ (absolutely sure that the face is old, i.e. has been studied during the encoding session). There were significant differences for old and new faces in all ratings except for rating ‘4’. ***b***, Subsequent memory performance based on the memory status (Item forgotten, Association forgotten, Association remembered) and the distraction condition (low and high distraction). There were no differences in performance between the different levels of distraction or memory status. Error bars represent SEM.

In the face recognition memory test, subjects were able to distinguish between old and new faces (2×6 ANOVA, interaction *F_(5, 80)_* = 32.94, *p*<0.001, Fig. 2a). Post-hoc paired-samples t-test revealed that subjects could not discriminate between old and new faces when giving a confidence rating of ‘4’ (*t_(16)_* = −0.05, *n.s.*). The proportion of trials with rating 1, 2 and 3 was significantly higher for new faces than for old faces (all *p*<0.005), and in rating 5 and 6 the reverse was true (both *p*<0.001). Therefore, old faces that received a ‘1’, ‘2’, or ‘3’ rating were defined as forgotten and old faces that received a ‘5’ or ‘6’ rating were defined as remembered, faces received rating ‘4’ were categorized as trials of “no interest” and excluded from further analysis. This definition is consistent with previous studies with similar design [14], [15]. Using these definitions we assessed the subjects' performance (number of remembered old faces divided by the total number of old faces) on the face recognition memory test. Memory performance in this test was clearly above chance level (one-sample t test, *t_(16)_* = 4.28, *p* = 0.001). Confidence ratings for old faces' recognition did not differ between the two levels of distraction (*F_(1, 16)_* = 0.76, n.s.).

Given our associative memory test with a forced choice design, low confidence yet correct responses might be based on exclusion or guessing. To prevent this from confounding our results we (conservatively) considered all trials that received a low confidence rating of ‘3’ as forgotten. Correct, confident answers (‘1’ or ‘2’ ratings) were defined as remembered, and incorrect confident answers (‘1’ or ‘2’ ratings) were defined as forgotten. Using these definitions the subjects' performance on this associative memory test for object-face pairs was calculated as the number of remembered trials divided by the total number of trials. Memory performance in this test was well above chance level (one-sample t test, *t_(16)_* = 11.72, *p*<0.001).

Based on the results of the face recognition memory test and the associative memory test, we assigned all trials to their memory status: Item forgotten, Association forgotten (item remembered), and Association (and item) remembered, trials in which subjects selected the incorrect face in the recognition memory task, but successfully retrieved the association were sorted into the category of “no interest” and excluded from further analysis. Performance in neither (status) bin appeared affected by the type of distraction task (see Fig. 2b, details are given in Table 1). Repeated measures 3×2 ANOVA revealed no main effect of distraction (*F_(1,16)_* = 1.13, *n.s.*), no main effect of memory status (*F_(2,32)_* = 0.16, *n.s.*), and no interaction between the two factors (*F_(2,32)_* = 0.40, *n.s.*).

**Table 1:** Mean number of trials (+SD) separated for the factors distraction and subsequent memory.

	Item Forgotten	Association Forgotten	Association Remembered	No Interest
Low Distraction	30.00	26.18	31.06	32.76
*SD*	*15.60*	*8.32*	*14.67*	*11.29*
High Distraction	30.06	27.00	29.76	33.18
*SD*	*12.07*	*8.60*	*16.45*	*11.89*

SD, Standard Deviation.

### Imaging results

#### Effect of distraction task

Imaging results for the distraction period showed a typical working memory activation pattern when contrasting the high with the low distraction condition. This contrast activated a set of brain regions including the bilateral middle frontal gyrus (BA 9), precuneus (BA 7), insula (BA 13), inferior occipital gyrus (BA 19) and fusiform gyrus (BA37), whole brain *p*(Family-Wise Error corrected)<0.05. No hippocampal activation was found, even at a liberal threshold (*p* = 0.01, uncorrected).

#### Non-associative memory and associative memory

Non-associative item memory formation, defined by the contrast Association forgotten (item recognized) minus Item forgotten, activated regions in the anterior medial temporal lobe including bilateral anterior hippocampus, amygdala, fusiform gyrus, right anterior parahippocampus as well as right middle hippocampus.

**Figure 3:**
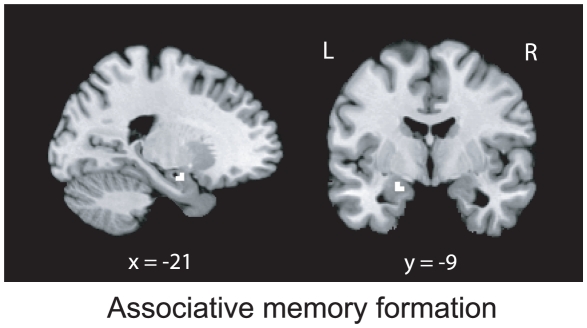
Brain regions activated in associative memory formation (Association remembered>Association forgotten). Sagittal view (***left***) and coronal view (***right***) show the activation in the left hippocampus (MNI -21 -9 -15, SVC, *p* = 0.029). Images are thresholded at *p*<0.001 uncorrected, for displaying purposes. L, left; R, right.

Assessing associative memory formation (Association remembered minus Association forgotten), a region in the hippocampus (see Fig. 3, local maximum at MNI -21 -9 -15, *p*(SVC) = 0.029) showed stronger activation during encoding when associative memory formation was successful compared to trials in which only item memory formation succeeded but associative memory formation failed. A region in the inferior frontal gyrus (BA 45; local maximum at MNI -56, 30, 12) showed a same subsequent memory effect with specific preference only in the high distraction condition (threshold at *p*(uncorrected)<0.001) but not in low distraction condition (even at a liberal threshold *p*(uncorrected)<0.01). The opposite contrast, which showed stronger activation when associative memory formation failed, revealed only one area located in the right parietal cortex (BA 40; local maximum at MNI 48, -54, 51; *p*(uncorrected)<0.001).

#### Interaction between distraction and associative memory formation

**Figure 4:**
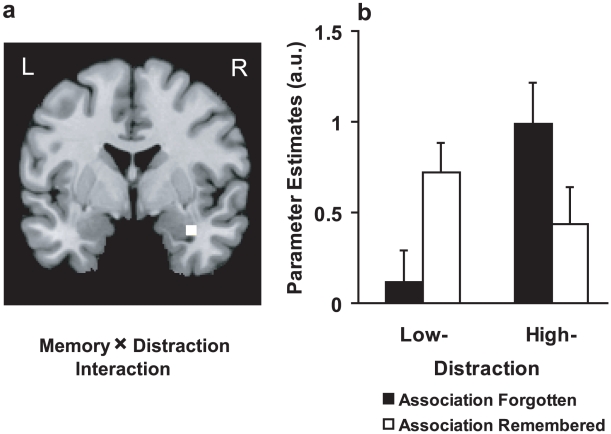
The interaction between distraction (low and high) and memory (Association forgotten and Association remembered). ***a***, An effect has been revealed in the right anterior hippocampus (MNI 33 -6 -21, SVC, *p* = 0.021). Image is thresholded at *p*<0.001 uncorrected, for displaying purposes. L, left; R, right. ***b***, We extracted the beta values from this region just to depict the direction of the interaction revealed. As can be seen, the interaction was based on a positive subsequent memory effect when distraction was low and a negative subsequent memory effect when distraction was high. Error bars represent SEM.

In the 2×2 interaction analysis between the memory status (Association forgotten and Association remembered) and the two distraction conditions, no brain region was found that exhibited a larger associative subsequent memory effect in the high compared to the low distraction condition [(Association remembered high distraction – Association forgotten high distraction)>(Association remembered low distraction – Association forgotten low distraction)], even when using a very liberal threshold of *p*(uncorrected)<0.05. However, the opposite interaction revealed a clear effect in the right hippocampus (see Fig. 4a, local maximum at MNI 33 -6 -21, *p*(SVC) = 0.021) extended into the amygdala. We extracted the beta values of this region to visualize the pattern of this interaction, and plotted the conditions according to their distraction level. As shown in Fig. 4b, this interaction appears to be based on a positive subsequent memory effect when distraction was low and a negative subsequent memory effect when distraction was high. Independent t-tests using SPM revealed that the positive subsequent memory effect on the low distraction level and the negative subsequent memory effect on the high distraction level are both significant (*p*(SVC) = 0.025 and *p*(SVC) = 0.009, respectively). When the association had been forgotten, the activation in the high distraction condition was significantly stronger than in the low distraction condition (*p*(SVC) = 0.002); however, when the association had been successfully remembered, the hippocampal activation showed no difference. These findings indicate that associative memory formation failed in the high distraction condition when too much hippocampal activity occurred and in the low distraction condition when too little hippocampal activity occurred. Thus, associative memory formation in the high distraction condition was only successful when activity was reduced sufficiently and in the low distraction condition when activity was increased sufficiently.

## Discussion

The present study revealed an interaction in hippocampal activity between the factors Associative memory formation and Distraction. Relatively lower hippocampal activation appeared related to better associative memory formation when encoding was embedded in a high distraction task, whereas a conventional, positive subsequent memory effect occurred in a low distraction task. This pattern of results occurred when the potentially interfering effect of the distraction task was generally compensated, because subsequent memory performance was unaffected by the difference in the distraction task. Nevertheless, the reason for failing to form a memory appears different between distraction conditions when analysis is trial-by-trial based. Too much noise might have impaired forming an associative memory trace in the high distraction condition. However, when ambient noise was reduced effectively, the remaining hippocampal activity was sufficient to form an associative memory trace successfully. In contrast, when ambient noise was low to start with, more, presumably task-relevant processing was beneficial for associative memory formation while low levels of hippocampal processing were related to subsequently forgotten associations. In sum, we obtained four data points on a bell-shape function relating hippocampal activity to the success of associative memory formation. There is, in line with our hypothesis, an optimal level of hippocampal activity for associative memory formation and sub- as well as supra-optimal levels that appear detrimental for associative memory formation.

Negative subsequent memory effects have been described before, although rarely in the medial temporal lobe, but in a number of neocortical regions including midline and lateral areas within the so-called default mode network [16]–[19]. These findings were interpreted as suggestive for inattention or mind wandering at study and, consequently, poor subsequent memory performance. These negative subsequent memory effects, however, were associated with positive effects in the medial temporal lobe and thus, our negative subsequent memory effect in the hippocampus cannot readily be attributed to default mode activity, although the hippocampus is sometimes regarded as part of this network [20]. However, the two ideas, increased default mode network activity or increased processing of task irrelevant information, are not mutually exclusive, because unconstrained processing of information related to the distraction task (i.e., noise) might be the basis for mind wandering and thus one can regard this additional activity as related to both noise and default mode processing. The facts that this additional activity can be reduced significantly when main task load was high [21], [22] supports our interpretation.

Our data suggests that noise reduction seem to be accomplished within the hippocampus. To achieve such noise reduction one has to assume a process that separates hippocampal representations of object-face associations from representations related to the distraction task. It is important to note that these representations, albeit quite different in their experimental characteristics, have large episodic overlap, because the entire context is identical. A process allowing such dissociation of overlapping representations might be pattern separation, which is known to enable distinct representations of overlapping input in the service of resolving interference [23]. Leutgeb and colleagues [24] have revealed a dual mechanism for pattern separation in which signals from the entorhinal cortex can be decorrelated both by changes in coincidence patterns and recruitment of non-overlapping cell assemblies in the hippocampus. However, it remains unclear whether successful pattern separation goes along with an increase [25], a decrease [26], [27], or no change in overall neural activity as measured with fMRI. Regardless, it has been shown that damage to a certain hippocampal subregion, the dentate gyrus, whose integrity is essential for normal pattern separation, affects selectively spatial memory acquired in a high spatial interference condition [28], indicating that pattern separation enables indeed ambient noise reduction when the study phase is submerged in distraction causing proactive interference.

While our data suggest a hippocampal process of noise reduction, they do not exclude alternative mechanisms for ambient noise reduction that might lead to less hippocampal input and hence to less overall processing as observed here. Negative subsequent memory effects could also reflect consequences of less input due to selective attention computed in inferior temporal regions [12]. Processing in extra striate visual cortex enables object-selective attention and thus forwarding of attended, task relevant information to the medial temporal lobe that lacks ambient noise related to unattended input [29], [30]. Alternatively, processes related to cognitive control computed in frontal regions could resolve competition among active representation [31], or suppress proactive interference [32], [33]. In line with the idea that the frontal lobe might have exerted control over hippocampal input, we detected an inferior frontal subsequent memory effect exclusively for the high distraction condition. However, no interaction between the factors Associative memory and Distraction occurred in this brain region. These alternative accounts are certainly valid, but our data does not provide evidence supporting them.

Despite the negative subsequent memory effect found here, the hippocampus plays nevertheless a critical role in associative memory formation. Here we probed a specific kind of associative memory formation, because the two constituents of each pair to be memorized were presented sequentially with a within-pair delay filled partly with a distraction task. Such discontiguous associative memory formation has been linked to medial temporal lobe activity previously [14], [15], [34], but it remained somewhat unclear whether this contribution was related to item maintenance during the within-pair delay [35] or final associative binding taking place after encountering the second constituent. The intervening distraction task, implemented here, that did not affect overall associative memory performance makes continuous maintenance in working memory less likely and thus, our data is supportive for a model in which the hippocampus supports the actual binding of the two constituents separated in time during memory formation [3], [36], [37]. This conclusion appears also closely in line with data obtained in classical conditioning experiments in which the hippocampus plays a critical role in trace conditioning only, where a delay period is included between the offset of the conditioned stimulus and the delivery of the unconditioned stimulus [38]–[42]. Thus, our data confirms the view that the hippocampus plays a critical role in associating discontinuous events during memory formation.

Our results appear to suggest that most effective associative memory formation is achieved at an intermediate level of activation in the right hippocampus while the left hippocampus shows the “classical” activity increase with successful associative memory formation. However, the two effects described in the left and right hippocampus were not significantly lateralized. Thus, they represent just significant effects in one medial temporal lobe, but there might have been the same, however, non-significant effects in the homologue area of the opposite hemisphere. Furthermore, the two effects are not found in overlapping, homologue areas. Thus, the two findings are do not support a hemispheric specialization and further studies will be needed to clarify this issue.

In sum, we provide initial empirical evidence for a bell-shape function relating hippocampal activity and success of associative memory formation. While intermediate levels of hippocampal activity appear optimal for associative memory formation too low levels and too high levels appear detrimental. Supra-optimal levels of hippocampal activity appear to occur when a distraction task, or a state of stress [12] interferes with memory formation presumably by adding noise to the task-relevant signal. If ambient noise reduction is sufficient, we can form coherent episodic memories across discontinuous events cluttered with irrelevant information, a situation confronted with every day.
